# Mouse models in the era of large human tumour sequencing studies

**DOI:** 10.1098/rsob.180080

**Published:** 2018-08-15

**Authors:** J. R. de Ruiter, L. F. A. Wessels, J. Jonkers

**Affiliations:** 1 Division of Molecular Pathology, The Netherlands Cancer Institute, Amsterdam, The Netherlands; 2 Division of Molecular Carcinogenesis, The Netherlands Cancer Institute, Amsterdam, The Netherlands; 3 Department of EEMCS, Delft University of Technology, Delft, The Netherlands; 4 Oncode Institute, Amsterdam, The Netherlands

**Keywords:** cancer, driver genes, mouse models, GEMMs, oncogenomics, genetic screening

## Abstract

Cancer is a complex disease in which cells progressively accumulate mutations disrupting their cellular processes. A fraction of these mutations drive tumourigenesis by affecting oncogenes or tumour suppressor genes, but many mutations are passengers with no clear contribution to tumour development. The advancement of DNA and RNA sequencing technologies has enabled in-depth analysis of thousands of human tumours from various tissues to perform systematic characterization of their (epi)genomes and transcriptomes in order to identify (epi)genetic changes associated with cancer. Combined with considerable progress in algorithmic development, this expansion in scale has resulted in the identification of many cancer-associated mutations, genes and pathways that are considered to be potential drivers of tumour development. However, it remains challenging to systematically identify drivers affected by complex genomic rearrangements and drivers residing in non-coding regions of the genome or in complex amplicons or deletions of copy-number driven tumours. Furthermore, functional characterization is challenging in the human context due to the lack of genetically tractable experimental model systems in which the effects of mutations can be studied in the context of their tumour microenvironment. In this respect, mouse models of human cancer provide unique opportunities for pinpointing novel driver genes and their detailed characterization. In this review, we provide an overview of approaches for complementing human studies with data from mouse models. We also discuss state-of-the-art technological developments for cancer gene discovery and validation in mice.

## 1. Introduction

Cancer is a disease in which normal cells are deregulated by disruption of their cellular processes, resulting in increased proliferation, survival and invasion of surrounding tissues. This disruption is generally attributed to mutations in so-called driver genes, which provide cells with a selective growth advantage and drive their malignant transformation. Broadly speaking, driver genes can be divided into two classes of genes: oncogenes and tumour suppressor genes (TSGs) [1]. Oncogenes drive tumour development when activated by mutations and typically promote cellular proliferation and/or survival [2], whereas TSGs are inactivated during tumourigenesis and are generally involved in processes protecting cells from DNA damage and malignant transformation.

**Figure 1.**
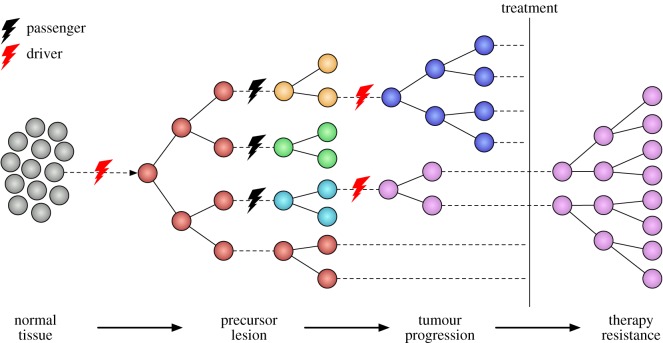
Darwinian evolution during tumour development. Tumourigenesis is a multi-step process in which initially healthy cells progressively acquire multiple mutations that disrupt their cellular processes and increase their tumourigenic potential. Although many of these mutations are passengers with no clear effect on the ability of cells to survive and proliferate, a few rare driver mutations may strongly increase the fitness of individual cells, allowing them to outcompete neighbouring cells. Over time, this stochastic process gives rise to Darwinian competition between subclones of cells harbouring different sets of mutations, driving selection towards subclones with increasing tumourigenic potential. External interventions such as drug treatments can influence this process by eradicating subclones that are sensitive to the given treatment. However, they can also drive selection towards subclones that are resistant to the treatment, leading to the emergence of therapy resistance as seen in many cancer patients.

Tumours are however not the product of single mutations, but develop progressively over time through the accumulation of multiple mutations. Depending on the affected genes, these mutations can increase the fitness or tumourigenic potential of cells (additional driver mutations), or have no clear contribution towards tumourigenesis (passenger mutations). Over time, this accumulation gives rise to subpopulations of cells (subclones) harbouring distinct sets of mutations, which are subject to Darwinian competition (clonal evolution) within the tumour lesion [3] (figure 1). This competition selects for further mutations, resulting in increased fitness and the continued evolution of competing subclones within the tumour. External influences such as the immune micro-environment or drug treatment can also strongly influence the evolutionary process, either by selecting for specific subclones that are intrinsically resistant to immune surveillance or treatment, or by applying additional evolutionary pressure to acquire new mutations that confer resistance [4].

Effective treatment of cancer patients is increasingly based on precision medicine, i.e. tailored treatments designed to target specific mutation-associated dependencies and/or vulnerabilities of a patient's tumour. It is therefore crucial to identify exactly which mutations contribute to tumourigenesis and how they do so. Although human sequencing studies have identified many genes contributing to cancer development, they do not provide evidence for causality or detailed insight into the biological mechanisms by which these genes drive tumour development. These studies also do not reveal whether drivers are essential for tumour maintenance and may therefore be of limited use for designing effective therapeutic strategies. In contrast, preclinical model systems such as genetically engineered mouse models (GEMMs) provide an experimentally tractable approach in which the biological effects of specific mutations can be studied in detail in a controlled genetic background. In this review, we describe several aspects of mouse models and how these can ultimately be applied to improve treatment of cancer patients. To this end, we first highlight several challenges in translating findings from human sequencing studies to the clinical setting, before explaining how some of these challenges can be addressed using complementary approaches in mouse model systems.

## 2. Challenges in human tumour sequencing studies

Several major human sequencing studies have been undertaken over the past years, aiming to identify and catalogue potential driver mutations across many different cancer types [5–8]. One of the key challenges in analysing data from these efforts has been the separation of driver mutations from passenger mutations. To address this issue, many computational approaches have been developed to select driver genes using signals of positive selection in the pattern of somatic mutations in genes across tumour samples. Examples include approaches based on mutation frequency [9,10], biases in the functional consequences of mutations [11–14] and clustering of mutations within genes [15].

Although these approaches have proven successful in identifying many driver genes affected by hotspot mutations, other types of mutations have proven more challenging. This is especially the case for DNA copy-number driven diseases such as breast cancer, in which approaches aimed at identifying recurrent copy number alterations typically identify regions harbouring many genes [16,17]. Similarly, complex genomic rearrangements [18] and mutations in non-coding regions [19] make it difficult to pinpoint specific target genes, requiring further prioritization of candidate genes using complementary approaches and/or exhaustive validation of potential drivers.

Besides the validation of putative driver genes, another important challenge is the further characterization of their biological roles. This insight is crucial for the identification of potential therapeutic opportunities. Currently, most large-scale studies perform no or limited *in vivo* validation of candidate genes [20–24], as this additional follow-up is typically time- and labour-intensive. Furthermore, although some studies do perform *in vitro* validation of candidate genes in human cell line models, this is likely to be of limited relevance [25] as cancer cell lines harbour many additional mutations and are grown in a highly artificial environment. Other *in vitro* models such as three dimensional (3D) tumour organoids [26,27] may provide an interesting alternative but also lack a tumour microenvironment and need to be grown in specific media [28], which may limit the clinical translatability of findings in these models.

Finally, many promising targeted therapies fail in the clinic due to the emergence of treatment resistance. To understand why this is the case, it is important to determine how different therapies impact the clonal evolution of a tumour and how this leads to the development of treatment resistance. These insights can then be used to develop new strategies that aim to prevent or overcome resistance. However, detailed studies of clonal evolution and treatment resistance are challenging as the development of resistance is often a stochastic process, as is evident from the observation that patients frequently develop multiple mechanisms of resistance to the same treatment [29–31]. Combined with the limited availability of pre- and post-treatment tumour samples from patients, this limits the potential of human studies for the analysis of resistance mechanisms. Identification and prediction of potential resistance mechanisms therefore requires experimental systems that allow us to quantify the range of expected resistance mechanisms for a given tumour and determine how these are impacted by different treatments or other factors such as diverse genetic backgrounds.

## 3. Experimental models of human cancer

### 3.1. Patient-derived models

Experimental models of human cancer should be easy to manipulate and recapitulate the genetic features and microenvironment of the original patient tumour as much as possible. Human cancer cell lines have often been used for cancer research, as these are derived from patient tumours and are easy to manipulate *in vitro*. However, human cancer cell lines are grown in a highly artificial environment and therefore undergo a stringent selection process when being established, resulting in homogeneous populations that no longer fully represent the genetic heterogeneity of the original tumour [32]. More recently, 3D organoid models have been developed to overcome this limitation by growing cells in three-dimensional media, which allows the formation of more realistic organ-like structures [33]. This technique has enabled the development of *in vitro* models for tissues that could not be established as cell lines [28], suggesting that organoids are subject to less evolutionary pressure and are therefore more likely to reflect the heterogeneity of the original patient tumours.

**Figure 2.**
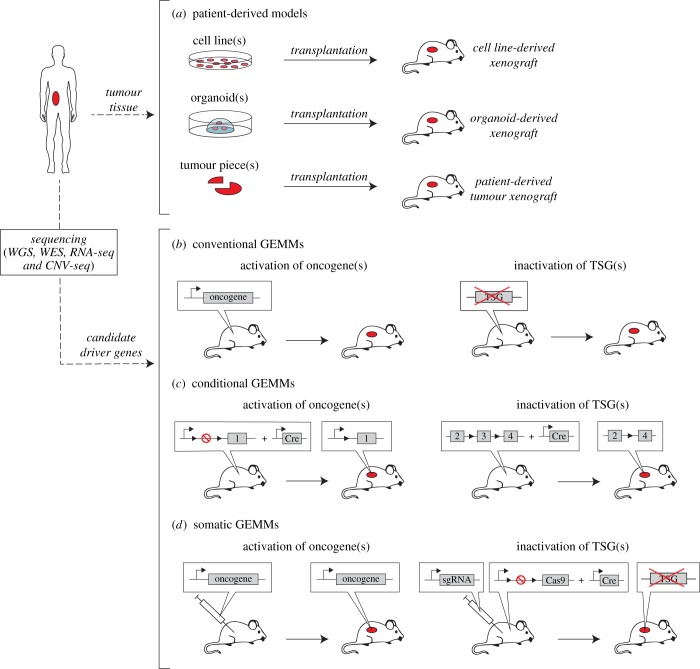
Schematic overview of different types of mouse models of human cancer. (*a*) Patient-derived models are created by transplanting human material into immune-deficient mice. This can be done by injecting either tumour-derived cell lines or tumour organoids, or by directly grafting human tumour pieces into mice. (*b*) In conventional GEMMs, de novo tumourigenesis is induced either by tissue-specific expression of an oncogene or by germline inactivation of a TSG. The engineered genes are typically selected based on pre-existing data from (human) sequencing studies. (*c*) In conditional GEMMs, de novo tumourigenesis is induced by tissue-specific inactivation of conditional TSG alleles and/or activation of conditional oncogenes via Cre-loxP-mediated recombination. (*d*) In somatic GEMMs, tumourigenesis is induced either by injecting lentiviral vectors expressing specific oncogene(s) into the tissue of interest, or by injecting Cas9 together with sgRNAs targeting specific TSGs. In the latter approach, Cas9 can also be expressed conditionally in the host mouse.

Human cell lines have been very popular in cancer research, as they remain relatively close to the human setting, while providing a convenient platform for studying cancer cell biology. As such, these *in vitro* models have proved instrumental in delineating key biological signalling pathways and in preclinical drug screening [34,35]. A drawback of human cell lines and organoids is, however, that they do not model interactions with the tumour microenvironment and the effects of angiogenesis and drug metabolism. To address these limitations, cell lines and organoids can be injected into immune-deficient mice (figure 2*a*) to create *in vivo* xenograft models. However, although these cell line-derived xenograft models do capture interactions between tumour cells and the (mouse) microenvironment, they do not recapitulate interactions with the immune system due to the use of immunocompromised mice.

An alternative approach is to directly transplant human tissue into immunodeficient host mice, thereby creating patient-derived tumour xenograft (PDX) models. Compared to cell line-based transplantation models, PDX tumours more faithfully retain the molecular, genetic and histological heterogeneity observed in the respective cancer patients, even after serial passaging in mice [36,37]. As such, PDX models have been a popular *in vivo* platform for preclinical drug screening in a large variety of cancer types, such as breast cancer [38,39], melanoma [29,40] and colorectal cancer [41–45]. However, drawbacks of PDX models are that certain tumour types are much harder to establish in mice than others, and xenografts may undergo mouse-specific tumour evolution [46]. Moreover, similar to cell line-based xenograft models, PDX models generally lack an active immune system and therefore do not capture interactions with the immune system.

Humanized mouse models aim to address this gap by engrafting components of the human immune system into otherwise immunocompromised mice [47,48]. One such approach is to engraft CD34^+^ human haematopoietic stem and precursor cells into the marrow of sublethally irradiated immunocompromised mice, allowing these cells to develop into a functional, humanized immune system [49]. Ideally, this engrafted immune system should mirror that of a human cancer patient, enabling detailed studies of interactions between tumours and the immune system. However, it currently remains challenging to faithfully recapitulate the full human tumour microenvironment in mice [48]. Moreover, accurate modelling of a patient's immune response requires the development of personalized xenograft models [50] in which both the immune system and implanted tumour tissue are derived from the same cancer patient.

### 3.2. Genetically engineered mouse models

A significant limitation of patient-derived models is that they are typically established using heavily mutated, end-stage tumours and can therefore not be used to study the effects of individual mutations on tumour initiation and progression. In contrast, genetically engineered mouse models (GEMMs) can be used to introduce individual mutations identified from human sequencing projects into a clean genetic background, allowing detailed characterization of these mutations and their effects on cancer susceptibility, tumour formation, progression and maintenance (table 1).

**Table 1.** Overview of mouse models and their characteristics.

model	de novo tumour formation	sporadic cancer model	immune competent	cancer gene validation	resistance mechanisms	main application(s)
PDXs	no	yes	no (partial in humanized models)	no	yes	therapy response and resistance
conventional GEMMs	yes	no	yes	yes	yes	superseded by conditional GEMMs
conditional GEMMs	yes	yes	yes	yes	yes	driver validation, modelling tumour progression
somatic GEMMs	yes	yes	yes (in Cas9 tolerant mice)	yes	yes	rapid driver gene validation, modelling tumour progression

The first GEMMs were developed by introducing cloned cancer genes into the genome of transgenic mice (figure 2*b*), providing the first conclusive evidence that mice could be made prone to developing tumours in a specific tissue by introducing transgenic expression of oncogenes such as *MYC*, *ERBB2* or mutant *KRAS* under control of a tissue-specific promoter [51]. Later, with the rise of gene-targeting technology, the effects of inactivating mutations in tumour suppressor genes (TSGs) such as *Trp53* or *Rb1* on tumour formation could be studied in knockout mice [52]. However, a significant limitation of these conventional GEMMs is that oncogenes are expressed in all cells of a particular tissue in transgenic mice, while TSGs in knockout mice are inactivated in all cells. In this respect, conventional GEMMs fail to mimic sporadic cancers, in which the accumulation of genetic events in a single cell results in tumourigenesis in an otherwise healthy organ.

To address this issue, conditional GEMMs were developed by employing somatic activation of oncogenes (e.g. *Bcr-Abl1*, *Erbb2*, *Myc*, *Hras* and *Kras*) and somatic inactivation of tumour suppressors (e.g. *Apc*, *Brca1*, *Brca2*, *Nf1*, *Nf2*, *Pten*, *Rb* and *Vhl*) (figure 2*c*) [53]. One of the most frequently used conditional strategies is the Cre/loxP recombinase system [54], in which (parts of) target genes are flanked by loxP recombinase recognition sites that recombine in the presence of Cre-recombinase to delete intervening DNA sequences. Using this system, oncogenes can be activated by removing engineered stop sequences that prevent gene expression in the absence of the recombinase, whereas TSGs can be inactivated by deleting exons that are crucial for gene function. Conditional GEMMs have been developed for a large variety of different cancers, generating a wealth of models that closely mimic the histopathological, molecular and clinical features of human tumours [55,56].

A limitation of conditional GEMMs is that generating new models is still time-consuming and expensive. Recent developments in somatic gene-editing techniques provide incredible potential to speed-up this process by allowing mutations to be introduced somatically into existing mouse models (figure 2*d*). Using these approaches, oncogenes can be introduced by injecting (viral) vectors expressing these gene(s) into the tissue of interest [57]. Similarly, TSGs can be inactivated using CRISPR/Cas9-mediated gene editing by injecting constructs containing Cas9 and single guide RNAs (sgRNAs) targeting the TSGs into Cas9-proficient mice [58]. Further developments such as CRISPR interference/activation [59] and CRISPR-based mutagenesis approaches [60,61] promise to further expand this toolkit, enabling the use of somatic engineering to rapidly model a wide variety of cancer-associated mutations in mice.

Another approach for upscaling experiments is to orthotopically transplant mouse tumour fragments into syngeneic mice, allowing further stages of tumour development to be studied in parallel [62]. A particular advantage of this strategy is that it enables flexible and rapid expansion of spontaneous tumours from specific genetic background(s), which can be used to study stochasticity in tumourigenesis and therapy response in advanced stages of the disease. Besides this, orthotopic transplantation models provide unique opportunities for studying metastatic disease by following metastatic progression after resection of the transplanted tumour fragment [63,64].

## 4. Identifying cancer drivers in mouse models

### 4.1. Mouse tumour sequencing

**Figure 3.**
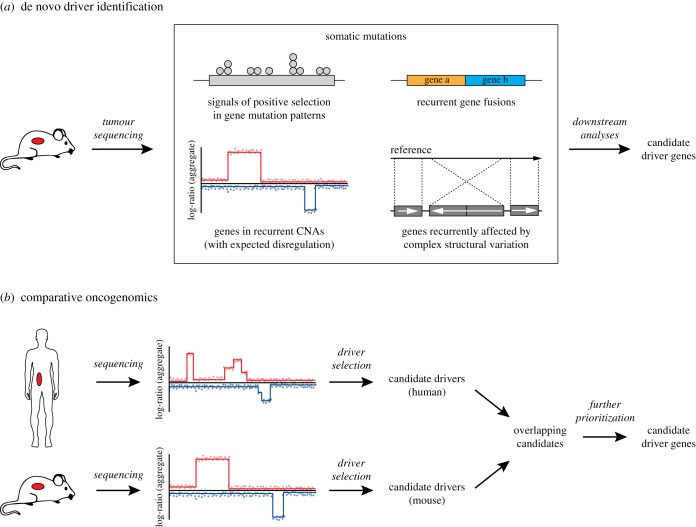
De novo driver gene identification in mice. (*a*) Driver genes can be identified in mice in the same fashion as for human tumours by using DNA/RNA-sequencing approaches aimed at identifying recurrent mutations, copy-number aberrations, gene fusions and complex structural rearrangements. (*b*) Comparative oncogenomics approaches allow refinement of candidate driver gene lists by focusing on genes that are recurrently mutated in both mouse and human tumours. In such an approach, candidates are typically first identified for both species individually, after which shared (orthologous) genes are selected and further prioritized based on existing knowledge or other data sources.

Besides candidate cancer driver analysis, mouse models can also be used to identify additional driver mutations by sequencing mouse tumours and identifying additional genes that are frequently mutated across tumours (figure 3*a*). Following this approach, driver mutations can be detected using various computational approaches, in the same fashion as previously described for human tumours. This can be particularly powerful when combined with mouse models of spontaneous tumour development, such as radiation- or carcinogen-induced [65–67] tumour models [68–70], which may more accurately reflect the heterogeneity and stochasticity of patient tumours than GEMMs. An additional advantage of using mouse tumour sequencing to identify driver genes is that, by sequencing tumours from GEMMs, we can specifically identify drivers that collaborate with the engineered driver mutations. As such, whole-exome and whole-genome sequencing approaches have been used to characterize the mutational landscapes of *Kras*-mutant mouse skin squamous cell carcinoma [71] and *Egfr*-, *Myc*- and *Kras*-driven lung cancers [72,73]. Similarly, whole-exome sequencing, RNA sequencing and DNA copy-number-based approaches have identified several driver genes in mouse models of *Brca1*- and *Brca2*-deficient breast cancer [74,75].

In general, mouse tumours tend to have lower mutational loads than their human equivalents. This reduced complexity offers easier interpretability, allowing for the extrapolation of critical genetic driver processes that may not be apparent in genetically complex human cancers [76], but may also be indicative of differences in tumour development between mouse and human tumours [77]. However, in some instances mouse tumours may harbour as many aberrations as human tumours, complicating the identification of driver genes. This is, for example, the case for tumours with high levels of genomic instability, such as *Brca1*/*Brca2*-deficient breast cancer models [74,75].

### 4.2. Comparative oncogenomics

A more powerful approach for identifying driver genes using sequencing approaches is to combine insights from mouse and human datasets and prioritize genes that are mutated in both species, as these are most likely to represent true driver genes. This can be done in an *ad hoc* setting, by identifying drivers of mouse tumours and comparing these with known mutations in human datasets, or as a deliberate strategy using comparative oncogenomics. In the latter approach, sequencing data from mouse and human tumours are typically first analysed to identify candidate driver genes for both species individually. These species-specific candidates are then integrated by only selecting genes and/or networks that are aberrated in both species (figure 3*b*). Remaining candidates can optionally be filtered using additional criteria, such as correlation with gene expression or prior knowledge from literature.

This comparative strategy has proven particularly effective for distinguishing driver genes from passengers in chromosomally unstable tumours. For example, in a mouse model of hepatocellular carcinoma (HCC), Zender *et al.* [78] identified a focal amplicon on mouse chromosome 9qA1, which was syntenic with amplifications in human HCCs on 11q22. Further filtering based on expression identified two drivers on this locus, *cIAP1* and *Yap*, which were shown to act synergistically in tumourigenesis. Similarly, copy-number sequencing of metastases from an inducible *Hras* model of a traditionally non-metastatic melanoma identified a focal amplification on mouse chromosome 16, which contained only eight candidate driver genes [79]. Further comparison with human *RAS*- and *MET*-driven melanomas identified a single gene, *NEDD9*, as the driver of these metastases. A recent study from Liu *et al.* [75] used whole-exome sequencing and RNA sequencing data from BRCA1-deficient mouse mammary tumours to identify multiple aberrations capable of activating the MAPK and/or PI3K signalling pathways. Similar mutations were identified in human triple-negative breast cancers (TNBCs), suggesting that inhibition of these pathways may be an effective therapeutic strategy for treating BRCA1-deficient TNBC.

## 5. Identifying drivers using forward genetic screening

Although additional driver genes can be identified by mouse tumour sequencing, this approach is not always optimal as mouse models can have a long tumour latency and may be more prone to acquire other types of mutations than those of interest (i.e. copy-number aberrations rather than point mutations or vice versa). Forward genetic screening approaches can address these issues by using various mutagenesis strategies to induce additional mutations and accelerate tumour formation, after which any tumours that developed can be studied to identify new drivers. The type of mutations that occur depends strongly on the type of mutagenesis that is being employed, meaning that different mutagenesis strategies can be used to specifically induce different kinds of mutations.

### 5.1. Screening using chemical mutagenesis

**Figure 4.**
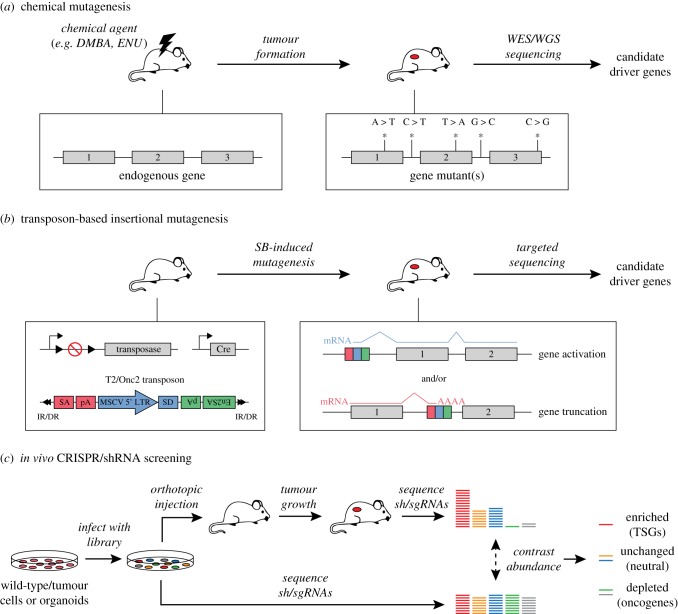
Schematic overview of forward genetic screening approaches. (*a*) In chemical mutagenesis approaches, mice are treated with a mutagenic compound and subsequently monitored for tumour formation. The resulting tumours can then be sequenced using whole-genome or targeted sequencing to identify mutations driving tumourigenesis. (*b*) In transposon-based insertional mutagenesis (TIM), tissue-specific expression of a transposase enzyme induces the mobilization of mobile elements called transposons, which can be re-integrated elsewhere in the genome. By doing so, transposons can result in the activation of oncogenes or inactivation of TSGs. IR/DR: inverted repeat/direct repeat sequences, SA, carp β-actin splice acceptor; pA, polyA; MSCV 5′ LTR, murine stem cell virus 5′ long terminal repeat; SD, splice donor; En2SA, mouse Engrailed 2 splice acceptor. (*c*) In *in vivo* shRNA/CRISPR screening approaches, cells or organoids are transduced with a library of lentiviruses encoding shRNAs or sgRNAs designed to target a set of genes. The transduced cells/organoids are then injected *in vivo*, after which the mice are monitored for tumour formation. Developed tumours are sequenced to determine the abundance of individual shRNAs/sgRNAs, which is contrasted to the starting population to identify if shRNAs/sgRNAs targeting specific genes are enriched (potential TSGs) or depleted (potential oncogenes and/or drug targets).

Chemical-based mutagenesis is one of the oldest mutagenesis strategies, in which cells or animals are treated with a chemical substance that damages the DNA and thereby induces mutations. The induced mutations are typically single nucleotide changes; however, the spectrum of these mutations depends on the used substance and can vary greatly between different chemicals. In *in vivo* chemical mutagenesis screens, animals (typically zebrafish or mice) are treated with a controlled dose of a chemical mutagen and subsequently monitored for tumour formation (figure 4*a*). Developed tumours can be sequenced using whole-genome or targeted sequencing strategies to identify mutations that may be driving tumourigenesis or metastasis [72,80].

An advantage of chemical mutagenesis compared to other mutagenesis approaches is that its bias towards single nucleotide changes makes it suitable for modelling the effects of human variants, which are often single point mutations that result in changes in the levels of expression or activity of a gene product. For this reason, chemical mutagenesis approaches have been used to mimic human mutational processes and to characterize the genomic landscapes of the mutational landscapes of mouse skin squamous cell carcinoma [71] and *Kras*-driven lung cancers [72]. However, when designing chemical mutagenesis screens, it is important to take the inherent mutational bias of chemicals into account. Currently, N-ethyl-N-nitrosourea (ENU) is a popular choice for chemical mutagenesis strategies aiming to model single-nucleotide variants (SNVs), as it results in a range of point mutations that mirrors the range of mutations observed in human tumours [81].

### 5.2. Screening using insertional mutagenesis

The bias towards point mutations also limits the utility of chemical mutagenesis strategies for modelling other types of mutations, such as increased gene expression resulting from gene amplifications. Insertional mutagenesis (IM) strategies provide an alternative approach, in which viral [82] or transposon [83–86] sequences are stochastically inserted into the genome, disrupting the expression of nearby genes (figure 4*b*). In transposon-based insertional mutagenesis (TIM) strategies, this process is mediated by a transposase enzyme, which excises transposons from a concatemer introduced in the genome of the mice and reintegrates them stochastically elsewhere. By placing the expression of this transposase under a tissue-specific promotor, mutagenesis can be restricted to specific tissues in the mouse.

The effects of insertions depend on the used transposon, but typically involve the activation of oncogenes using promotor sequences and/or inactivation of tumour suppressors by truncating genes. For example, the *T2Onc/2* transposon, which is frequently used in Sleeping Beauty IM screens [83,84], contains enhancer/promotor (MSCV) and splice donor (SD) sequences that allow the transposon to initiate transcription and drive the (over-)expression of nearby genes. The *T2Onc/2* transposon also contains two splice acceptor sites (SA/En2SA) combined with a bi-directional polyA sequence, which allow the transposon to truncate transcripts if integrated within a gene. Depending on the gene and the relative location of the insertion, these truncations can inactivate genes by resulting in an unstable transcript or inactive protein, or activate genes by removing inhibitory protein domains [87,88].

A considerable advantage of IM strategies is that insertions can be specifically captured via PCR amplification before sequencing, enabling cheap and efficient retrieval of the insertion sites compared to genome-wide sequencing. A drawback of transposon-based systems is that they generally show some bias in terms of their insertion patterns, due to sequence integration biases or biases towards specific gene features (e.g. gene bodies or promotors) [89]. For this reason, screens using different transposon systems (such as the Sleeping Beauty [83,84] or PiggyBac [85,86] systems) may identify different candidate genes, even if screens are performed in the same genetic background. Moreover, target genes are typically identified using windows around the insertion sites [89,90], which may lead to the identification of false-positive candidate genes.

Despite these drawbacks, TIM and other mutagenesis systems have been valuable for identifying cancer-associated driver genes in mouse models of a large variety of cancer types, including breast cancer [91–93], melanoma [94], hepatocellular carcinoma [95] and gastric cancer [96]. Additionally, as mutagenesis remains constitutively active in these models, TIM has also been used to identify drivers of metastasis formation [97] and acquired resistance to drug treatments [98,99]. Finally, new computational approaches based on RNA-sequencing data have been developed to improve target gene prediction and offer additional insight into the effects of insertions on the expression of the affected gene [100,101].

### 5.3. shRNA screening

In contrast to the previously described non-targeted genome-wide screening approaches, library-based screening approaches, such as loss-of-function screens based on RNA interference (RNAi) technology, can be used to target specific sets of genes. In pooled RNAi screening approaches, cells are transduced with lentiviruses encoding short hairpin RNAs (shRNAs) targeting specific genes that, when integrated into the genome, result in stable and heritable suppression of the corresponding gene [102]. To perform an *in vivo* RNAi screen, (tumour) cells transduced with a lentiviral shRNA library can be injected orthotopically in animals [103], which are subsequently monitored for tumour growth (figure 4*c*). Once developed, tumours are harvested and sequenced to quantify the frequency of each shRNA in the tumour cell population. By comparing these frequencies to those of the initial starting population, this approach can identify which shRNAs are enriched in the tumour and are therefore likely targeting TSGs whose loss is beneficial for tumourigenesis. Conversely, depleted shRNAs may identify potential oncogenes and/or genes that are crucial for tumour maintenance.

The scope of shRNA screens depends entirely on the used library, meaning that screens can be designed to target all genes in a genome-wide fashion or to test a small number of pre-selected candidate genes. As such, shRNA screens can not only be used to identify novel driver genes, but can also be used for narrowing down lists of potential drivers or to validate putative driver genes. Compared to *in vitro* approaches, *in vivo* shRNA screens provide the opportunity to expose the vulnerabilities of tumour cells in the context of their microenvironment and can be used to study drivers of metastasis and therapy resistance [104–106]. Drawbacks of shRNA screening include variable efficiency between shRNAs in the knockdown of their respective target genes [107] and off-target effects [108]. Successful *in vivo* shRNA screens have been reported for a variety of cancer types, including xenograft models of hepatocellular carcinoma [109], lymphoma [110,111], leukaemia [112,113] and glioma [114,115].

### 5.4. CRISPR screening

With the development of CRISPR-based technologies, it has also become possible to perform pooled loss-of-function screens by inactivating genes using CRISPR/Cas9*-*mediated genome editing. CRISPR/Cas9-based loss-of-function screens are generally performed in the same fashion as shRNA screens by transducing cells with pools of single guide RNAs (sgRNAs) targeting different genes, injecting the transduced cells *in vivo* and contrasting the abundance of sgRNAs in tumours with their abundance in the starting population [116–118]. However, in contrast to shRNA screening, gene editing via CRISPR/Cas9 disrupts the genes by DNA cleavage and thereby introduces insertions/deletions in their genomic sequence, resulting in frameshifts that induce heterozygous or homozygous knockout of genes rather than a reduction in expression.

Compared to shRNA screens, CRISPR-based screens have been reported to be remarkably efficient and suffer less from off-target effects than shRNA screens [119,120]. CRISPR-based screening approaches are less amenable to studying dosage-dependent effects as genes are inactivated rather than transcriptionally suppressed, although dosage reduction can be achieved if Cas9 only induces heterozygous loss of the gene, as we have previously observed for *in vivo* validation of candidates from a *Sleeping Beauty* IM screen [93]. With the development of new technologies, CRISPR-based screening approaches are extending beyond loss-of-function screens by enabling gene activation using CRISPRa [121], gene inhibition using CRISPRi [122] and the introduction of point mutations with dCas9-AID or dCas9-APOBEC base editors [60,61].

## 6. Validating and characterizing candidate driver genes

**Figure 5.**
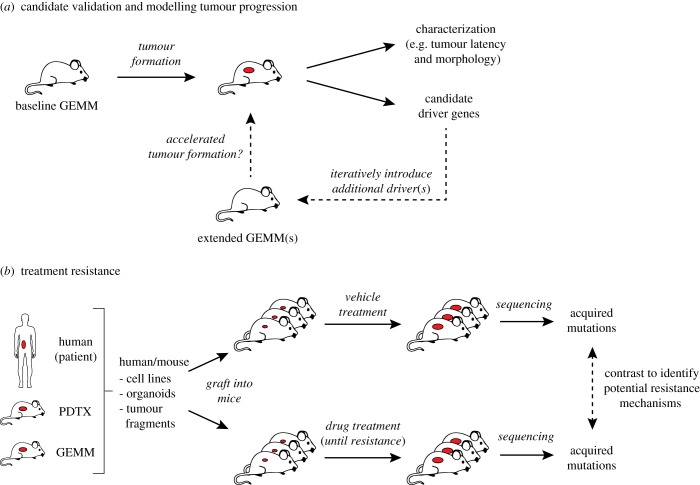
Validating candidate cancer genes and studying therapy resistance. (*a*) Candidate cancer driver genes can be validated *in vivo* by developing mouse models containing the observed mutations and monitoring the mice for tumour formation. Developed tumours can be characterized to determine if they reflect the expected phenotype(s) and sequenced to identify additional candidate driver genes. These additional candidates can be introduced into the same baseline GEMM model to determine their effect on tumourigenesis. By applying this process iteratively, this type of approach can be used to unravel the contribution of different cancer genes during various stages of tumourigenesis. (*b*) Mouse models can also be used to identify mechanisms of acquired therapy resistance by transplanting tumour-derived cell lines, tumour organoids or tumour pieces into multiple recipient mice and subjecting these mice to different treatments. Tumours that become resistant to treatment can then be sequenced and contrasted to vehicle-treated tumours to identify acquired mutations driving the resistance phenotype.

After identifying putative driver genes, it remains important to verify that these genes actually contribute to tumourigenesis. In many studies, human tumour cell lines are used for this purpose by studying the effects of perturbations in candidate driver genes on tumour cell growth. However, as previously described, these *in vitro* models suffer from several drawbacks, including the presence of additional mutations and the lack of a tumour microenvironment. An alternative approach is to introduce candidate driver mutations into an established GEMM of the cancer type in which the mutations were identified, in order to study their effect on tumourigenesis (figure 5*a*). These additional mutations can be introduced using either germline approaches (such as the GEMM-ESC strategy [123]) or somatic approaches (e.g. via injection of viral vectors), as described in previous sections.

Once established, the resulting mouse models can be studied to determine how the additional mutations affect tumour incidence, latency and/or metastasis compared to the baseline mouse model. The new models can also be used to study the effects of the extra mutations on therapy response. Any developed tumours can be studied in detail to determine how the additional mutations affect tumour morphology and the tumour (immune) microenvironment. Additionally, by using sequencing strategies or screening approaches to identify additional driver genes in these more complex GEMMs, mutational landscapes can be compared between models with different drivers to determine how additional drivers affect the evolution of tumours initiated by the engineered mutations and if this provides clues to any driver-specific vulnerabilities.

In particular, CRISPR/CAS9-based somatic cancer modelling approaches enable rapid *in vivo* testing of (combinations of) candidate cancer genes and have been used to validate driver genes for a wide range of cancer types, including breast cancer [58], colorectal cancer [124], pancreatic cancer [125] and liver cancer [126]. Additionally, multiplexed somatic engineering methods provide the opportunity to rapidly validate multiple candidate genes at the same time, while simultaneously studying Darwinian selection between the different candidates and how this selection is influenced by cellular/tissue contexts and pre-existing mutations [126,127]. Finally, using iterative approaches, drivers can be identified and introduced progressively into mouse models of increasing complexity. This type of approach can be used to study tumour formation and progression in detail and establish the contributions of different driver genes at various stages of tumourigenesis [128].

## 7. Studying drug response and treatment resistance

Ultimately, knowledge of driver genes and their effects is used to develop novel therapeutic strategies that target specific vulnerabilities of the tumour, enabling effective treatments with minimal side effects. Following this premise, personalized therapies are generally designed by either targeting identified driver genes directly (if possible) or by targeting other genes in the same signalling pathway. A well-known example is *BRAF*-mutant melanoma, which is targeted by inhibiting the mutant BRAF kinase and/or MEK, a protein downstream of BRAF in the RAS/MAPK signalling pathway. Alternatively, tumours can be targeted therapeutically by exploiting a synthetic lethality resulting from the driver mutation(s). A classic example of synthetic lethality is poly(ADP-ribose) polymerase (PARP) inhibition in BRCA-deficient tumours, which specifically targets cells with defects in their homologous recombination (HR) pathway due to loss of HR factors such as BRCA1 and BRCA2.

Before moving into the clinic, drugs are generally first tested for anti-cancer efficacy in a preclinical setting, either using *in vitro* models (cell lines, organoids) or *in vivo* models (xenograft models, GEMMs, PDXs). To identify which treatments are most effective in different cancer types or tumours with different genetic backgrounds, several efforts have been made to set up large biobanks of PDX models for high-throughput drug screening purposes [39,42,129,130]. By correlating treatment sensitivity with sequencing data from the same tumours, these approaches can also be used to identify genetic markers of intrinsic (pre-existing) therapy resistance. An example of this approach has been given by Bertotti *et al.* [42], who identified HER2 amplification to be driving resistance in a subset of cetuximab-resistant colorectal PDX tumours and showed that combined inhibition of HER2 and EGFR induces overt, long-lasting tumour regression.

Besides intrinsic therapy resistance, many targeted therapies fail in the clinic due to the emergence of drug resistance which is acquired during treatment. As such, a key challenge for improving the efficacy of these therapies is to identify (and ideally pre-empt) (epi)genetic changes that underlie this acquired treatment resistance. Both GEMMs and PDXs can be used to identify potential *in vivo* resistance mechanisms by grafting cell lines, organoids or tumour fragments into multiple recipient mice, which are then subjected to different treatments (figure 5*b*). Upon relapse, resistant tumours can be sequenced and compared with tumours from vehicle-treated mice to identify possible resistance mechanisms. Using this approach, our laboratory identified several resistance mechanisms to PARP inhibitor treatment in mouse models of *BRCA1*- and *BRCA2*-deficient breast cancer [131–133]. This type of approach can also be combined with various mutagenesis strategies, for example by using insertional mutagenesis to induce resistance and identify potential resistance mechanisms [98,99,134].

Besides identifying potential resistance mechanisms, an important challenge is to determine how resistance actually arises and to design therapeutic strategies accordingly. For example, in cases where tumours acquire additional (epi)genetic changes during treatment (as described above), therapies should ideally be designed to pre-empt and prevent the most likely paths of resistance. On the other hand, cases where resistance is driven by pre-existing sub-populations of intrinsically resistant cells [135] will require different treatment strategies. Traditionally, studying intra-tumour heterogeneity has been challenging with bulk-sequencing technologies. Single-cell sequencing approaches [136,137] promise to revolutionize these analyses by providing detailed insight into the (transcriptional) heterogeneity of tumour cells, enabling the identification of sub-populations of cells that may be driving resistance [138]. Furthermore, approaches such as lineage tracing can be used to track the dynamics of tumour evolution, providing detailed insight into which cell populations expand and contract during treatment. As such, lineage tracing-based approaches have been used to identify origins of resistance in mouse models of squamous cell carcinoma [139], prostate cancer [140] and mouse intestinal adenomas [141].

## 8. Conclusion and future perspectives

**Figure 6.**
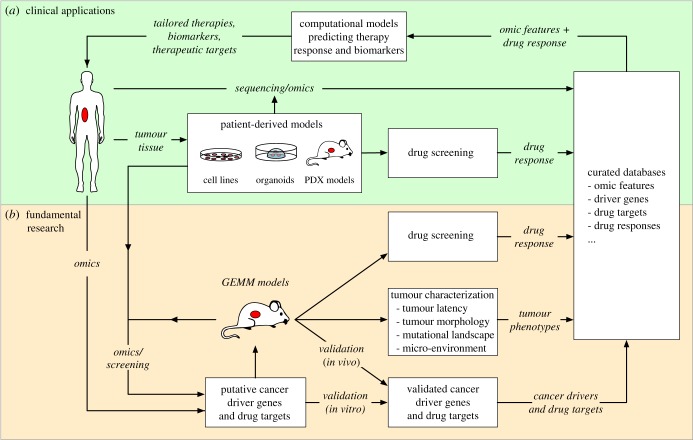
The roles of model systems in designing personalized combination therapies for effective cancer treatment. This figure illustrates how human/mouse model systems are ultimately used to identify and characterize cancer driver genes in different types of cancer, and how these insights can be used to inform patient treatment. This process can essentially be divided into two parts: clinical application and fundamental research. (*a*) On the clinical side, patient tumours can be analysed (sequencing/omics) to identify mutations and potential cancer drivers. In parallel, tumour material can also be used to establish patient-derived model systems, which can be used to screen for drug efficacy and study treatment resistance. By combining drug response data with mutations identified through sequencing, this approach can be used to train computational models predicting optimal therapies and identify biological features explaining the observed drug response. (*b*) On the fundamental side, mouse models can also be used to identify cancer drivers (through sequencing and genetic screening) and for drug screening. In contrast to patient-derived models, GEMMs can also be used to conclusively validate cancer driver genes *in vivo* and to perform detailed dissection of how different driver genes affect tumour development and progression. This information can be used for predictive models in the clinic, but can also be used to formulate new hypotheses and additional experiments, fuelling further fundamental research into the molecular underpinnings of cancer.

The success of personalized anti-cancer therapies hinges on how accurately we can predict whether a given patient tumour will respond to a given treatment, allowing clinicians to select the most effective therapeutic strategy for treating a patient. Ideally such an approach would be implemented by feeding omics data and other data types (e.g. imaging, pathology) from patient tumours into (computational) models that predict which therapies are most likely to be effective based on specific tumour biomarkers (figure 6). Creating such models requires detailed insight into which mutations are driving tumour development and how these affect therapy response. Combined with high-throughput drug screening approaches, *in vitro* and *in vivo* model systems provide crucial platforms for assessing sensitivity to different therapies across multiple cell lines or tumours, enabling the construction of correlative models that predict the efficacy of these treatments for new tumours. More detailed genetic modelling in (mouse) model systems can further refine these correlative models by providing causative evidence that combinations of mutations drive cancer development and/or affect therapy response. In addition, mouse modelling enables detailed characterization of the effects of drivers on other tumour phenotypes, such as tumour latency, morphology, mutational landscape and interactions with the (immune) microenvironment. Genetically engineered mouse models also provide powerful platforms to critically evaluate new candidate drug targets [142] and thereby improve the robustness of preclinical cancer target identification [143].

Modelling of human cancer using genetically engineered mice is complicated by the observation that tumours generally contain multiple driver lesions, which can strongly influence their sensitivity to treatments targeting specific drivers. As a result, accurately assessing therapy response may require the generation of complex mouse models containing multiple driver genes that are frequently encountered together in a given type of cancer. Using germline engineering approaches, generating models with multiple driver lesions has been challenging due to the extensive breeding and animal husbandry involved. Somatic approaches using CRISPR/CAS9-based gene editing and over-expression vectors alleviate this bottleneck, by providing the technology to quickly create new mouse models by introducing different combinations of mutations into a pre-existing baseline mouse model. The rapidly ongoing refinement of these tools will further expand the repertoire of mutations that can be modelled in this manner, enabling the rapid creation of new models reflecting the mutations observed in patient tumours, which can be used to test the effects of novel therapeutic strategies targeting these mutations.

Mouse models are of particular interest for studying immune-based therapies, which have recently garnered much interest due to their high efficacy in treating advanced stages of several cancer types [144–147]. However, responses to immune therapies have been highly variable, with some patients responding well to treatment while others exhibit severe side effects [148,149]. This indicates that a better understanding of the complex interactions between the immune system and tumour cells is needed for continued improvement in immune-based therapies, which cannot be studied using *in vitro* models or xenograft models lacking an active immune system. As such, immune-competent mouse models such as GEMMs are better suited for studying interactions between tumours and the immune system, although care should be taken to ensure that used models reflect the variety of genetic and other factors that may influence an immune response. Additionally, differences in the mouse and human immune systems may limit the relevance of GEMMs, requiring the use of humanized mouse models to accurately model immune responses in human patients.

Besides designing novel therapies, other important clinical challenges include identifying which patients are most at risk of developing cancer and should be screened for preventative treatments. Although factors such as genetic background and lifestyle have been shown to have a profound influence on cancer risk and survival, our insights into how these factors influence cancer development is still limited. Due to their tightly controlled genetics, mouse models are uniquely suited for examining the effects of genetic backgrounds and how these interact with specific driver genes in an *in vivo* setting. Similarly, mouse models may also be used to model the effects of specific lifestyles (e.g. diet, gut microbiome, circadian rhythm, exposure to mutagens) on cancer risk and development [150]. Combined, insights from such models will hopefully allow us to incorporate knowledge of genetic modifiers and lifestyle influences into clinical tests that improve the identification and clinical management of individuals at high risk for cancer.

Improved screening imposes its own challenges, as population screening programmes identify many early lesions that will not necessarily progress to cancer and therefore do not actually require treatment. Unfortunately, in many cases it is currently not possible to distinguish which lesions are indolent and which will progress to invasive cancer, leading to overdiagnosis and overtreatment [151]. Identifying which tumour cell-intrinsic and -extrinsic factors contribute to tumour progression will hopefully provide important insight into the tumourigenic process and allow us to develop tests that distinguish between high- and low-risk lesions. These studies will require models that allow us to study the early stages of cancer, which is not possible using end-stage tumour material from patients. GEMMs can provide a powerful platform for this type of research, as lesions in these models can be studied at any stage during de novo tumour development. Moreover, by introducing mutations identified in pre-malignant lesions from patients, mouse models can be used to determine the contributions of these mutations to tumour initiation and progression, and to screen for additional factors that may be required for malignant transformation.

Tumour progression, metastasis and escape from therapy are phenomena which are driven by intra-tumour heterogeneity [152]. Single-cell sequencing approaches provide a particularly promising approach for studying tumour progression by enabling detailed characterization of distinct cell populations within a given tumour [136,137]. Combined with (CRISPR-based) lineage tracing [153,154], single-cell approaches in GEMM tumours can be used to study the early dynamics of pre-malignant lesions and determine which cell populations play a role in driving eventual tumourigenesis. Similarly, longitudinal sampling of mouse tumours may be used to determine how cell populations within tumours evolve during tumour progression and under therapeutic pressure, providing insight into how certain (epi)genetic changes may drive tumour evolution and the development of therapy resistance. Finally, detailed characterization of non-transformed cells within the tumour—such as cancer-associated fibroblasts and tumour-infiltrating immune cells—can be used to explore the complex interactions between tumour cells and the (immune) microenvironment [155,156] and how these interactions change during tumour progression or during therapy stress.

It is important to keep in mind that tumours arising in GEMMs of human cancer may not necessarily reflect all characteristics of human tumours. For example, GEMM tumours may contain lower numbers of somatic mutations compared to the cognate human tumours [73] and fewer mutations seem to be required for cancer formation in mice compared to humans [157]. As such, it remains important to establish whether mouse tumours accurately reflect the relevant aspects of the cognate human cancers, in terms of histopathology, mutational landscape and transcriptional profile. Additionally, due to their limited genetic heterogeneity, it is unrealistic to expect that mouse models will sufficiently represent the heterogeneity of patient populations. It will therefore remain crucial to combine findings from GEMMs with information from other sources, including sequencing data from patient populations and experimental data from other model systems, such as (human) tumour organoids and PDX models.

Finally, efforts to collect and catalogue mouse sequencing data have been relatively limited compared to efforts involving human sequencing studies. To fully exploit the large compendium of mouse sequencing and screening data, it will be important to collect these data in centralized repositories and create portals to query these data, allowing researchers to quickly explore existing datasets and compare tumour characteristics across different mouse models. Fortunately, several efforts are already underway to collect data in application-specific databases [158], to create portals visualizing data from PDX models and to adapt software like cBioPortal [159] for visualizing tumour data from non-human organisms. We expect that these initiatives will play an important role in disseminating insights from mouse models and improve accessibility for cross-pollination with human sequencing efforts.
